# Editorial: Prevention of Abuse and Harassment in Athletics and Sports

**DOI:** 10.3389/fspor.2021.801060

**Published:** 2021-12-02

**Authors:** Stéphane Bermon, Paolo Emilio Adami, Toomas Timpka, Mike Hartill

**Affiliations:** ^1^World Athletics, Health and Science Department, Monaco, Monaco; ^2^LAMHESS, Université Côte d'Azur, Nice, France; ^3^Athletics Research Center Linköping University, Linköping, Sweden; ^4^Department of Social Sciences, Edge Hill University, Ormskirk, United Kingdom

**Keywords:** abuse, harassment, sports, perpetrators, governance

The Research Topic on Abuse and Harassment in Athletics and Sports recently published in Frontiers aimed at providing athletes, their entourage, and sport governing bodies with new data and concepts about a concern of growing importance. Indeed, following the numerous campaigns to publicly denounce acts of abuse and harassment in the civil society or working environment, the world of sport has also brought its share of scandals and revelations in recent years.

In their epidemiological report on young elite Athletics athletes, Bermon et al. confirmed that the observed lifetime prevalence of verbal, physical and sexual abuses in their studied population is very similar to those reported in recreational athletes and in the general population. Inside Athletics setting, verbal abuse, an often-neglected form of abuse, was frequently reported in both male and female athletes (21–23%). Although some geographical discrepancies were noted, prevalence of physical (12 vs. 9%) and sexual (12 vs. 7%) abuses were on average slightly higher in male than in female elite athletes, respectively. Another interesting finding is the higher prevalence of touching and penetrative sexual abuses in young male athletes compared to young female athletes. Contrary to a common belief, athletics coaches were identified as perpetrators of all these forms of abuse in only 25% of cases; friends, partners and other athletes together representing 58% of the perpetrators of abuses committed in the athletics setting.

In their scoping review, Gaedicke et al. carefully analyzed the coach-athlete relationship and sexual violence. Imbalance of power appeared as a crucial concept favoring the occurrence of sexual or physical abuses. In most countries and sports, these coach positions are still held by men who often develop an authoritarian coaching style, which is believed to be associated with better sports results. However, this coaching style has been identified in many studies as a risk factor for the emergence of violence. The closeness between a coach and an athlete is also identified as a complex component which has been described as a positive factor in the coach–athlete relationship, an important aspect of sporting success, and a risk factor for the emergence of sexual violence. Setting *a priori* clear boundaries in the coach-athlete relationship, clearly defining coach's and athlete's roles and mutual expectations (including a written document or a contract) are effective ways of preventing abuses and maintain trust. The case of a love relationship between a coach and an athlete is an extreme but not rare case. Even in the case of an *a priori* consensual relationship, raises the problem of the value of the consent given by an immature athlete in a situation of emotional dependence or under the effect of an asymmetry of power in the relationship. Finally, as described by Bermon et al.; Gaedicke et al., the phenomenon of heteronormativity must be considered with caution when studying abuse and violence in sports. Indeed, recent statistics show that same-sex abuse is more frequent than assumed and even more often hidden by the victims.

In an attempt to better define the characteristics of perpetrators responsible for child sexual abuse in sports, Vertommen et al. analyzed the casefiles of sixteen perpetrators of child and teenagers sexual abuse in sports to assess static and dynamic risk factors related to sexual recidivism. There was not a single distinctive profile of the perpetrators and the authors noted that most of them were socially integrated and well-respected within the sport organization where they often had responsibilities in the sport organization. These findings reiterate the importance of general primary and secondary prevention in sport and society at large.

From a sport governing body perspective, it is recommended that beyond the physical and sexual abuse and harassment, a global culture of “Safe Sport” or safeguarding is developed. This culture should for instance consider topic like doping (Guo et al.) or concussion (Malcolm), or new trends in sports like e-sport and their health consequences (Kelly and Leung). Designing and implementing such a “Safe Sport” program is a huge challenge for sport governing bodies. Indeed, it is a multi-faceted approach requiring human and financial resources which are often lacking in small to medium size sports federation or clubs. Among the necessary steps to be completed by governing bodies, education on safeguarding, designing, and implementing policies or rules, developing an independent channel for the victims to report abuses or misbehaviours, and triggering investigations, have been reported as being the most important ones (Gurgis and Kerr). In these policies and regulations design, general counsels with expertise in human right, legal aspects and ethical compliance have an important role to play (Carska-Sheppard and Ammons).

To achieve all goals previously listed, Komaki et al. suggest a paradigm shift: from wrongdoing to right doing and from punishment to reward. Reinforcing initiative accentuating a positive culture is considered as a promising solution. For instance, recruiting and promoting sport coaches with a culture of supporting athlete well-being should be experimented.

The present Research Topic on Abuse and Harassment in Athletics and Sports sheds further light on both the variety and complexity of the problem of verbal, physical and sexual abuses in sport. Although much epidemiological and sociological work is still needed, it invites those responsible for the prevention and eradication of abuses in sport to be very open-minded in their understanding and treatment of this problem affecting all areas of our societies.

## Author Contributions

All authors listed have made a substantial, direct, and intellectual contribution to the work and approved it for publication.

## Conflict of Interest

The authors declare that the research was conducted in the absence of any commercial or financial relationships that could be construed as a potential conflict of interest.

## Publisher's Note

All claims expressed in this article are solely those of the authors and do not necessarily represent those of their affiliated organizations, or those of the publisher, the editors and the reviewers. Any product that may be evaluated in this article, or claim that may be made by its manufacturer, is not guaranteed or endorsed by the publisher.

